# Subclinical vasculopathy and skeletal muscle metrics in the singapore longitudinal ageing study

**DOI:** 10.18632/aging.203142

**Published:** 2021-06-07

**Authors:** Shir Lynn Lim, Xiao Liu, Qi Gao, Shwe Zin Nyunt, Lingli Gong, Josephine B. Lunaria, Carolyn SP Lam, Arthur Mark Richards, Shiou Liang Wee, Lieng Hsi Ling, Tze Pin Ng

**Affiliations:** 1 Department of Cardiology, National University Heart Center, Singapore; 2 Geriatric Education and Research Institute, Singapore; 3 Gerontology Research Programme, Department of Psychological Medicine, Yong Loo Lin School of Medicine, National University of Singapore, Singapore; 4 Department of Medicine, Yong Loo Lin School of Medicine, National University of Singapore, Singapore; 5 Department of Cardiology, National Heart Center, Singapore; 6 Cardiovascular and Metabolic Disorders Program, Duke-NUS Graduate Medical School Singapore, Singapore; 7 Cardiovascular Research Institute, National University Heart Center, Singapore; 8 Christchurch Heart Institute, University of Otago, New Zealand; 9 Faculty of Health and Social Sciences, Singapore Institute of Technology, Singapore

**Keywords:** muscle mass, muscle function, aortic stiffness, carotid stiffness, endothelial function

## Abstract

Frailty is associated with future cardiovascular events in older adults. This cross-sectional study examined the relationship between subclinical vasculopathy with measures of skeletal muscle mass and function. Asymptomatic community-dwelling Asians ≥55 years underwent assessments for subclinical vasculopathy (carotid intima-media thickness (cIMT), aortic and carotid stiffness, and endothelial function), muscle mass (calf circumference adjusted for body mass index) and function (knee extension strength, 6-meter fast gait speed). Multivariable regression analyses for associates of muscle mass/function controlled for demographics and cardiometabolic risk factors. Among 336 participants (median age 62 years, 55.1% male, 3.6% sarcopenia), cIMT, aortic and carotid stiffness inversely correlated with muscle mass, strength and gait speed; cIMT remained independently associated with gait speed (β=-0.26) in multivariable analyses. Age and sex significantly modified the relationship between subclinical vasculopathy and muscle mass/function. Associations, only found in those aged ≥70, included cIMT with gait speed (β=-0.48) and knee strength (β=-9.33), and aortic augmentation index and aortic stiffness composite z-score with gait speed (β=-0.11 and β=-0.19 respectively). Among males, cIMT correlated with gait speed (β=-0.31). The association of subclinical vasculopathy with skeletal muscle mass and function in asymptomatic adults ≥55 years is best reflected by cIMT. The roles of mediating pathways deserve further evaluation.

## INTRODUCTION

Recent literature has highlighted the important association between frailty and its precursor syndrome, sarcopenia, with cardiovascular disease (CVD), and cardiovascular and all-cause mortality [1–3]. The pathobiology underlying this association is incompletely understood; age-related chronic inflammation, common cardiometabolic risk factors and age-related changes in vascular thickening and stiffness, permeability and vasomotor tone impairing skeletal muscle function, may be involved [1, 4].

The few studies that have explored potential pathophysiological relationships between markers of sarcopenia and preclinical vascular disease have yielded conflicting findings. While several observational studies show independent associations between arterial stiffness with skeletal muscle decline [5] and cross-sectional thigh muscle area, [6] others have reported no association of sarcopenia with cardiometabolic risk or carotid intima-media thickness (cIMT) [7]. Notably, several studies focus solely on an imaging as opposed to any functional metric of sarcopenia, and the majority do not comprehensively evaluate preclinical CVD or cardiometabolic risk factors. Furthermore, sarcopenia is identified and characterized in patients who already manifest signs and symptoms, despite increasing recognition of its earlier development [8].

In this study, we prospectively examined a population-based sample of multi-ethnic Asian adults without overt CVD to determine the association of alterations in vascular structure and function, features which precede established CVD as intermediate phenotypes, with muscle mass and function. We hypothesized that multiple indices of systemic and regional vascular remodeling and dysfunction are associated with skeletal muscle metrics, possibly explained by shared cardiometabolic risk factors or other age-related factors.

## RESULTS

Baseline characteristics are summarized in Table 1. Study participants had a median (inter-quartile (IQR)) age of 62 (59-67) years, were mostly males (55.1%) and Chinese (82.1%). Cardiometabolic risk factors were highly prevalent – 54.2% had hypertension, 11.9% diabetes mellitus (DM), 73.5% dyslipidemia and 53.7% central obesity. Only 12 participants (3.6%) fulfilled the Foundation for the National Institutes of Health (FNIH) Sarcopenia Project criteria for sarcopenia, of whom 9 were at least 70 years of age [9].

**Table 1:** Characteristics of study participants.

**Variable**	**Participants(n=336)**
**Demographics**	
Age in years, median (IQR)	62.0 (59.0-67.0)
Body mass index in kg/m^2^, median (IQR)	24.8 (22.2-27.2)
Sex, n (%)	
Male	185 (55.1)
Female	151 (44.9)
Ethnicity, n (%)	
Chinese	276 (82.1)
Malay	44 (13.1)
Others	16 (4.8)
Housing type, n (%)	
1-2 room	42 (1.6)
3-5 room	243 (73.0)
High end public/ private housing	48 (14.4)
**Cardiometabolic risk factors**	
Smoking status, n (%)	
Non-smoker	264 (78.8)
Former smoker	46 (13.7)
Current smoker	25 (7.5)
Hypertension, n (%)	182 (54.2)
Diabetes, n (%)	40 (11.9)
Dyslipidemia, n (%)	247 (73.5)
Central obesity, n (%)	180 (53.7)
**Vascular Markers, median (IQR)**	
Structural atherosclerosis	
cIMT (mm)	0.7 (0.6-0.8)
Carotid stiffness	
AC (mm2/Kpa)	0.7 (0.6-0.9)
Ep (Kpa)	117.6 (92.1 – 149.5)
β-index	8.9 (7.2-11.2)
cAIx (%)	21.5 (13.5-29.9)
cPWV (m/s)	6.6 (5.8-7.4)
Composite z-score	-0.1 (-0.4-0.4)
Aortic stiffness^^^	
cfPWV (m/s)	8.6 (7.5-10.0)
aAIx (%)	36.0 (31.0-41.0)
aPP (mmHg)	50.0 (42.0-60.5)
Composite z-score	-0.1 (-0.4-0.4)
Endothelial function	
RHI	2.2 (1.8-2.5)
**Skeletal muscle metrics, median (IQR)**	
Gait speed (m/s)	1.4 (1.2-1.7)
Knee strength (kg)	17.3 (14.0-22.3)
Calf circumference/BMI	1.4 (1.3-1.5)
**Primary Outcome**	
Sarcopenia, n (%)	12 (3.6)

Median (IQR) for all continuous variables, and number (percentage): for all categorical variables.

Central obesity, waist circumference≥90 for male and ≥80 for female; carotid stiffness composite z-score : average of (Zβ-index + ZcAIx + ZcPWV), aortic stiffness composite z-score: average of (ZcfPWV + ZaAIx + ZaPP).

^Indices of aortic stiffness, where arterial path length was measured

Abbreviations: IQR, inter-quartile range; BMI, body mass index; cIMT, carotid intima-medial thickness; AC, arterial compliance; Ep, elastic modulus; β, beta; cAIx, carotid augmentation index; cPWV, carotid pulse wave velocity; cfPWV, carotid-femoral pulse wave velocity; aAIx, aortic augmentation index; aPP, aortic pulse pressure; RHI, reactive hyperemia index; Calf circumference/BMI, calf circumference adjusted for body mass index.

### Univariate analyses (Table 2)

Significant inverse associations were observed between cIMT, indices of carotid stiffness (pressure-strain elasticity modulus (Ep) and β-index), aortic stiffness (carotid-femoral pulse wave velocity (cfPWV), aortic augmentation index (aAIx), aortic pulse pressure (aPP) and aortic stiffness composite z-score) with gait speed. Carotid arterial compliance (AC) was positively associated with knee strength; carotid stiffness composite z-score and some indices of aortic stiffness (aAIx and aortic stiffness composite z-score) were negatively associated with knee strength. Multiple markers of carotid stiffness (Ep, carotid pulse wave velocity (cPWV) and carotid stiffness composite z-score) and aortic stiffness composite z-score were inversely associated with calf circumference adjusted for body mass index (BMI) (calf circumference/BMI), a marker of muscle mass. Reactive hyperemia index (RHI) was not associated with muscle mass and function in univariate analyses.

**Table 2:** Univariate associations between subclinical vasculopathy and skeletal muscle metrics (n=336).

		**Gait speed**			**Knee strength**			**Calf circumference/BMI**	
		**β**	**p**		**β**	**p**		**β**	**p**
Atherosclerosis	cIMT	-0.37	*<0.001		-1.74	0.379		0.03	0.601
Carotid stiffness^#^									
	AC	-0.00	0.979		2.39	*0.027		0.05	0.105
	Ep	-0.00	*0.007		-0.01	0.133		-0.00	*0.007
	β-index	-0.01	*0.011		-0.19	0.058		-0.01	0.067
	cAIx	0.01	0.517		-0.44	0.216		-0.01	0.329
	cPWV	-0.03	0.054		-0.31	0.309		-0.03	*0.002
	Composite z-score	-0.05	0.059		-1.05	*0.045		-0.04	*0.004
Aortic stiffness^^^	cfPWV	-0.04	*0.036		-0.48	0.186		-0.02	0.139
	aAIx	-0.04	*0.038		-1.07	*0.003		-0.01	0.221
	aPP	-0.04	*0.021		-0.63	0.078		-0.02	0.105
	Composite z-score	-0.08	*0.001		-1.57	*0.003		-0.03	*0.033
Endothelial function	RHI	-0.01	0.772		-1.25	0.086		0.00	0.900

*Statistically significant at p<0.05.

^Indices of aortic stiffness, where arterial path length was measured.

#Indices of carotid stiffness calculated using derived central aortic pressures.

Abbreviations as in Table 1.

Findings were fairly consistent for cfPWV calculated using the arterial path length formula, [10] and summarized in Supplementary Table 1.

### Multivariable analyses (Table 3)

**Figure 1:**
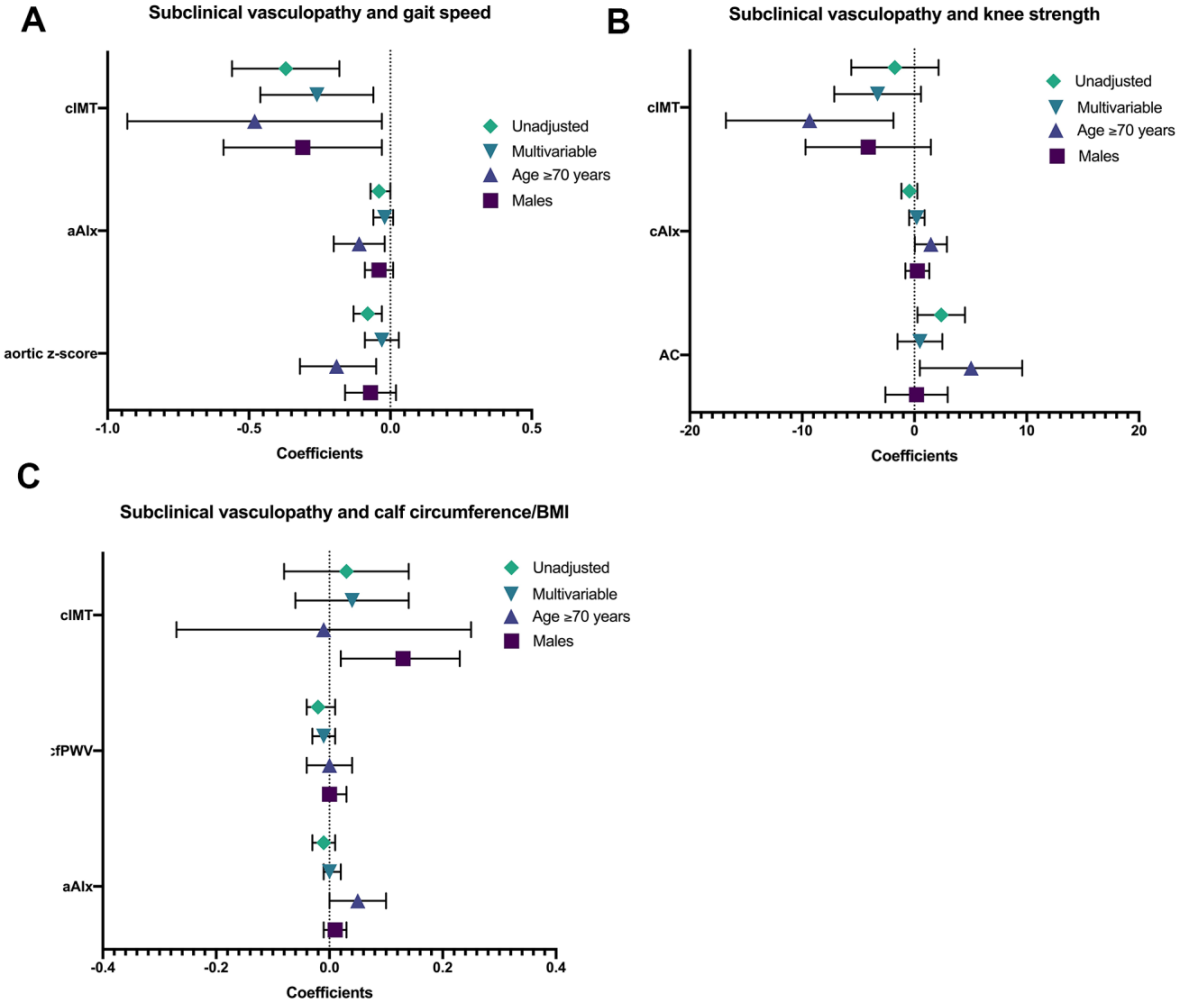
shows the univariate, multivariable, age- (≥70) and sex-stratified (males) associations between subclinical vasculopathy with (**A**) gait speed, (**B**) knee strength, and (**C**) calf circumference/BMI. *AC calculated using derived central aortic pressures. Abbreviations: Calf circumference/BMI, calf circumference adjusted for body mass index; cIMT, carotid intima-medial thickness; aAIx, aortic augmentation index; cAIx, carotid augmentation index; AC, arterial compliance.

The strength and significance of the associations were attenuated after adjustment for demographics and cardiometabolic risk factors. cIMT remained inversely associated with gait speed (Figure 1). None of the markers of subclinical vasculopathy were independently related to knee strength and calf circumference/BMI.

**Table 3:** Multivariable linear regression of subclinical vasculopathy and skeletal muscle metrics (n=336).

		**Gait speed**			**Knee strength**			**Calf circumference/BMI**	
		**β**	**p**		**β**	**p**		**β**	**p**
Atherosclerosis	cIMT	-0.26	*0.011		-3.28	0.096		0.04	0.390
Carotid stiffness^#^									
	AC	-0.03	0.618		0.49	0.632		-0.01	0.695
	Ep	-0.00	0.362		0.00	0.859		0.00	0.861
	β-index	-0.02	0.348		-0.06	0.853		0.00	0.605
	cAIx	0.01	0.408		0.21	0.545		0.00	0.984
	cPWV	-0.01	0.722		0.05	0.892		-0.00	0.914
	Composite z-score	-0.01	0.783		0.17	0.741		0.01	0.839
Aortic stiffness^^^	cfPWV	0.01	0.885		-0.59	0.131		-0.01	0.382
	aAIx	-0.02	0.239		-0.19	0.591		0.00	0.620
	aPP	-0.01	0.455		0.11	0.751		-0.00	0.984
	Composite z-score	-0.03	0.284		-0.46	0.409		-0.00	0.869
Endothelial function	RHI	-0.01	0.807		-0.64	0.336		0.02	0.306

Multivariable analysis, accounting for demographics (age, sex) and cardiometabolic risk factors (smoking, central obesity, diabetes, hypertension and dyslipidemia).

*Statistically significant at p<0.05.

#Indices of carotid stiffness calculated using derived central aortic pressures.

^Indices of aortic stiffness, where arterial path length was measured.

Abbreviations as in Table 1.

There was a significant age interaction in the associations between aortic stiffness with gait speed (cfPWV and aortic stiffness composite z-score), and with calf circumference/BMI (aAIx and aortic stiffness composite z-score) (Supplementary Table 2). Age was also a significant effect modifier in the association between RHI and calf circumference/BMI.

Significant sex interaction was seen in the associations between cIMT with knee strength and calf circumference/BMI (Supplementary Table 2). Sex was a significant effect modifier in the associations between aortic stiffness (aAIx and aortic stiffness composite z-score) with knee strength, as well as aortic stiffness (aortic stiffness composite z-score) with calf circumference/BMI.

The small number of sarcopenic patients precluded a sub-group analysis.

Findings using derived aortic stiffness indices are summarized in Supplementary Table 1.

### Age-stratified analyses(Supplementary Table 3)

Age-stratified multivariable analyses were performed to investigate the associations between indices of vascular health with muscle mass and function in participants aged ≥70 and <70 years, and presented in Supplementary Table 3. Among participants ≥70 years old, cIMT was inversely associated with both gait speed and knee strength (Figure 1). Markers of aortic stiffness, aAIx and aortic stiffness composite z-score, were inversely associated with gait speed. There was an independent and positive association between cAIx with knee strength. In contrast, there were no significant associations between markers of subclinical vasculopathy with muscle mass and function in participants <70 years old.

Findings using derived aortic stiffness indices are summarized in Supplementary Table 1.

### Sex-stratified analyses (Supplementary Table 4)

Sex-stratified multivariable analyses were performed to investigate the associations between indices of vascular health with muscle mass and function in male and female participants, and presented in Supplementary Table 4. Among male participants, cIMT was inversely associated with gait speed and positively associated with calf circumference/BMI (Figure 1). Among female participants, cfPWV, a marker of aortic stiffness, was inversely associated with calf circumference/BMI, a relationship which showed near-significance (p=0.050).

Findings using derived aortic stiffness indices are summarized in Supplementary Table 1.

## DISCUSSION

Our community-based study of middle-aged and older Asian adults without overt CVD found that intermediate phenotypes of CVD were significantly associated with measures of muscle mass and function. The strength of these associations was substantially reduced after adjusting for demographics and cardiometabolic risk factors, supporting their mediating roles in this relationship. In older adults ≥70 years of age, independent associations between cIMT and aortic stiffness with gait speed, and between cIMT with knee strength remained. In males, cIMT remained independently associated with gait speed. These directionally plausible associations of vascular biomarkers with muscle function amongst older participants support the notion that subclinical cardiovascular disease is associated with phenotypic frailty.

The association between CVD and muscle mass and strength in older persons is complex but appears more than coincidental, involving commonly occurring and shared mediating pathways that interact with each other in a bidirectional fashion over time. We found an independent negative association between cIMT and gait speed among our participants, consistent with existing literature showing similar associations between cIMT with gait dysfunction [11–13]. In age- and sex-stratified analyses, this association was significant in older adults ≥70 years of age and males. Potential explanations for these observations include: 1) slow gait speed reflects the functional consequences of vascular aging, 2) slow gait speed could be a marker of poor physical fitness, a well-known CVD risk factor, 3) hypertension is both a major determinant of cIMT [14] and risk factor for cerebral white matter hyperintensities, which are associated with reduced gait speed, [15] and 4) subclinical vasculopathy and decline in skeletal muscle function may become more apparent beyond age 65 [16].

Besides the inverse association of knee strength in participants ≥70 years of age with cIMT, there was also a positive association with cAIx. This counter-intuitive association could be spurious as: 1) the association with knee strength was borderline significant in multivariable but clearly non-significant in univariate analyses, suggesting over-adjustment by covariates in the model, and 2) cAIx plateaus after age 55 and may be a less reliable marker of arterial stiffness in older subjects [17].

Compared to the aggregate index of aortic stiffness (composite z-score), the association between carotid stiffness and skeletal muscle markers was less robust. Both the aorta and carotid artery are elastic vessels, which stiffen with aging and hypertension. Yet, the ultrastructure of these vessels is dissimilar. The aorta is more sensitive to aging particularly in the presence of cardiometabolic risk factors, [18] due to factors such as increased cross-linked collagen peptides in the aortic media, reduction in blood flow through the vasa vasorum and activation of the renin-angiotensin-aldosterone system with increase in levels of angiotensin II. The accelerated aging of the aorta compared to carotid artery may explain its sensitivity as a marker of subclinical CVD. Indeed, aortic stiffness is an independent predictor of all-cause and cardiovascular mortality and cardiovascular events in patients with hypertension, [19] diabetes mellitus, [20] end-stage renal failure [21] and in elderly subjects [22] while data are less consistent for arterial stiffness measured at other arterial sites [23]. Thus, aortic stiffness and carotid stiffness should not be used as interchangeable predictors.

Endothelial function is a general barometer of vascular health and endothelial dysfunction precedes frank atherosclerosis. Contrary to some studies, we did not find any association between RHI, a purported measure of endothelial function, and skeletal muscle metrics. A recent cohort study showed a significant correlation between RHI and skeletal muscle strength (measured by hand grip) in 236 older, rural-dwelling Korean women [24] while a very small study in older American subjects found significant independent associations between RHI and muscular power but not strength [25]. Compared to our subjects, those enrolled in these two studies were older and/or had greater activity limitation. Moreover, RHI reflects alterations in flow and digital microvascular dilatation that are not purely nitric oxide-dependent, and may not be a simple index of endothelial function [26].

We found age to be a significant interaction term in our analyses - the persistent association between subclinical vasculopathy and markers of skeletal function amongst older participants suggests the additional roles of age-related factors, notably chronic inflammation and neuroendocrine dysregulation. Aging is associated with a pro-inflammatory state, and population-based studies suggest an important role for inflammation in sarcopenia [27–29]. In addition, the insulin resistance, and reductions in Type 2 fast-twitch muscle fibres and satellite cells associated with aging lead to loss of muscle mass and subsequent infiltration with fat and adipose tissue [30]. Metabolically active fat depots could sustain sarcopenia and worsen insulin resistance by inducing a chronic inflammatory state [27]. Data on inflammatory markers in our study population were unfortunately not available.

Our analyses also showed sex to be a significant interaction term. In males, cIMT independently associated with gait speed, and positively associated with calf circumference/BMI. Whereas in females, we noted an inverse association between aortic stiffness with calf circumference/BMI which was near-significant. Calf circumference is a surrogate of muscle mass, and our findings are in keeping with prior studies using thigh computed tomography which suggest that arterial stiffness is an independent risk factor for age-related decline in muscle mass [5, 6]. We accounted for lower skeletal muscle mass in females, using separate cut-offs for calf circumference as a marker of muscle mass in males and females. Sex-specific differences in the prevalence and risk factors of frailty have been described, [31] and it is possible that arterial stiffness exerts a greater influence on muscle mass in females compared to males. The positive association between cIMT with calf circumference/BMI in males is counter-intuitive; the application of calf circumference/BMI as a surrogate of muscle mass in our population deserves further study.

The present study is one of a few community-based studies investigating the association of subclinical vasculopathy with skeletal muscle metrics in generally asymptomatic middle-aged to older adults (≥55 years old). We excluded subjects with a history of myocardial infarction, heart failure or other cardiovascular diseases, thus eliminating a major source of confounding. The low prevalence of defined sarcopenia in our younger (relative to other studies) [5–7] free-dwelling participants, works to advantage in aiding understanding of the complex relationships between vascular disease and premorbid decline in muscle mass and function. Despite this low prevalence, we could still discern significant correlations between skeletal muscle metrics and a swathe of structural and functional vascular markers. Other strengths included comprehensive phenotyping of arterial structure, biophysical properties and responsiveness. Limitations include the relatively small sample size, and the cross-sectional study design which precluded inferences about causality. Our ability to assess the association between endothelial function and skeletal muscle metrics was limited by the use of RHI, which reflects both nitric oxide-dependent and independent pathways. Finally, we lacked data on circulating biomarkers of chronic inflammation which could help to clarify the association of subclinical vasculopathy with muscle mass and function among subjects aged ≥70 which was not entirely explained by cardiometabolic factors.

## CONCLUSIONS

Our results support a link between systemic vascular health and skeletal muscle mass and function in middle-aged and older Asian adults. This association may be best reflected by cIMT given its independent association with muscle strength and function, principal determinants of sarcopenia, in older adults. The role of mediating pathways and temporal relationships in the development of subclinical vasculopathy in frail persons should be examined in a larger longitudinal study.

## MATERIALS AND METHODS

### Study setting and subjects

Study participants were community-dwelling middle-aged to older adults identified from the second wave of the Singapore Longitudinal Ageing Study (SLAS-II), an ongoing community-based cohort study of aging and health transitions among 3200 adults 55 years and older in Singapore [32]. The study sample consisted of a subset of 336 participants free of myocardial infarction, heart failure, or stroke, who underwent comprehensive vascular profiling and participated as control subjects in the Singapore Heart Failure Outcomes and Phenotypes (SHOP) study [33]. The study was approved by the Institutional Review Board, National University Singapore, and all participants gave written informed consent.

### Cardiometabolic risk factors

Dyslipidemia was defined as raised low-density lipoproteins level (≥3.4 mmol/L), [34] or self-reported dyslipidemia. Hypertension was defined as systolic BP ≥140 or diastolic BP ≥90 mm Hg, [35] or self-reported hypertension. Diabetes mellitus was defined as raised fasting plasma glucose (≥ 7 mmol/L), or self-reported type 2 diabetes mellitus. Central obesity was defined as waist circumference ≥90 cm for men and ≥80 cm for women.

### Vascular indices

All subjects underwent an array of non-invasive tests of vascular structure and function, several of which are known to predict adverse cardiovascular events and mortality [36–38].

### Carotid intima-media thickness (cIMT)

cIMT was determined by high resolution B-mode ultrasound using a 10.5MHz UST-5412 linear transducer and Prosound α10 system (Hitachi Aloka Medical Ltd., Tokyo, Japan) in accordance with guidelines of the American Society of Echocardiography [39]. The common carotid artery (CCA) was scanned in anterior, posterior and lateral planes on both left and right sides. cIMT was measured 1cm proximal to the carotid bulb, in an area free of plaque. IMT measurements in all planes were averaged, and the mean of both right and left IMT used for analysis.

### Conduit arterial stiffness

The aortic pressure waveform is made up of a forward pressure wave, created by left ventricular ejection, and a reflected pressure wave, with the timing and amount of reflection determined by aortic stiffness and arteriolar tone [40]. The speed of forward wave transmission, a reproducible measure of stiffness, is measured as carotid-femoral pulse wave velocity (cfPWV) and the prematurity and magnitude of wave reflection from the terminal aorta quantitated by the aortic augmentation index (aAIx) [40].

### Carotid-femoral pulse wave velocity (cfPWV)

Using the SphygmoCorVx system (AtCor Medical, West Ryde, NSW, Australia), applanation tonometry was performed to determine cfPWV. cfPWV was calculated as the carotid to femoral arterial path travel distance divided by the transit time. This distance was obtained by subtracting carotid-sternal notch distance from sternal notch-femoral distance (shortest distances, ignoring body contour). Transit time was obtained by subtracting the time between onset of the electrocardiographic R wave and the foot of the carotid pulse (averaging 8-10 sequential waveforms). Left and right-sided measurements were obtained for each patient and averaged; the mean of left- and right-sided measurements were used for analysis. Left and right-sided cfPWV were additionally calculated for each patient using a population-derived formula for arterial path length measured directly by magnetic resonance angiography [10]. The means of left and right-sided calculated values were used for analysis.

### Aortic augmentation index (aAIx) and aortic pulse pressure (aPP)

The SphygmoCorPx system uses applanation tonometry to obtain the radial artery waveform and applies a transfer function to convolve radial to aortic pressure [41]. aPP was the difference between the systolic and diastolic pressures of the central aortic pressure waveform. aAIx was calculated as the increment in pressure from the first systolic shoulder of the ascending aortic pressure wave to the peak of the second, late systolic shoulder, expressed as a percentage of the aortic pulse pressure (aPP) [42].

### Local arterial stiffness

Local stiffness of the CCA was quantified using the eTRACKING method on a Prosound α10 ultrasound system (Hitachi-Aloka Medical Ltd., Tokyo, Japan) [43]. Using radiofrequency signals, eTRACKING detects motion of opposed CCA walls in real-time, to 0.01mm resolution at 10MHz. The following indices were calculated from ensemble-averaged waveforms, using derived central aortic systolic and diastolic BP:

(i) *Arterial compliance (AC)* or the ratio between variations in arterial cross-sectional area and pulse pressure, as π(Ds×Ds - Dd×Dd) / [4(Ps- Pd)] where Ps = systolic BP, Pd = diastolic BP, Ds = maximum vessel diameter and Dd = minimum vessel diameter.

(ii) *pressure-strain elasticity modulus (Ep)* which expresses compliance relative to initial vessel diameter, as (Ps - Pd) / [(Ds - Dd)/Dd].

(iii) *β-index*, a relatively blood pressure-independent parameter of stiffness, as ln (Ps/Pd) / [(Ds - Dd)/Dd].

(iv) One-point PWV (cPWV).

(v) *Carotid augmentation index (cAIx),* determined from the CCA waveform, similarly to aAIx.

We converted selected raw values of indices of carotid and aortic stiffness into standardized Z-scores, which were summed to produce two composite arterial stiffness scores: aortic stiffness z-score=average of (ZcfPWV + ZaAIx + ZaPP) and carotid stiffness z-score=average of (Zβ- index + ZcAIx + ZcPWV).

### Endothelial function

Endothelial function includes vascular permeability and vasomotor tone, which are regulated by the expression, activation, and release of nitric oxide and other bioactive substances. This was assessed by fingertip peripheral arterial tonometry (PAT) using the EndoPAT device (Itamar Medical, Caesarea, Israel) [44]. Participants underwent the test while supine in a quiet, temperature-controlled room following standardized pre-test preparations, including abstinence from food and exercise for 12 hours, coffee or tea for 24 hours, alcohol and cardioactive medications for 48 hours prior. With arms positioned at the level of the heart, PAT probes were placed on bilateral index fingers, with one acting as the ‘study” finger and the other as control. After the baseline recording, arterial flow in the arm ipsilateral to the study finger was occluded for 5 minutes using a rapid cuff inflation system (Hokanson E20 and AG101, D.E. Hokanson Inc., Bellevue, WA, USA) to 60 mmHg above systolic blood pressure or 200 mmHg, whichever higher. PAT signals were recorded for at least 5 minutes following deflation. A *reactive hyperemia index (RHI)* was calculated as the ratio of reactive hyperemic response (average amplitude of the PAT signal 90-150s after cuff deflation) to basal flow (average PAT amplitude over 3.5 min), indexed to the contralateral control arm and multiplied by a proprietary baseline correction factor. RHI ≤1.67 was used as the threshold value for “endothelial dysfunction”.

### Outcomes measures

The clinical outcomes of interest were adapted measures of low lean mass plus reduced muscle function which define sarcopenia, as per the FNIH Sarcopenia Project [9].

### Skeletal muscle metrics

### Muscle mass

According to FNIH criteria, low lean mass is defined as appendicular lean body mass (ALM)/ BMI <0.789 in men and <0.512 in women [9]. In the absence of DXA, we used calf circumference instead of ALM [45, 46]. Calf circumference at mid-calf level of both legs was measured in cm, and mean values of the dominant leg used. To account for individual body build, we adjusted calf circumference using BMI, and used separate cut-offs for males and females when using calf circumference/BMI as a marker of muscle mass. In the SLAS-II cohort, we estimated low muscle mass by identifying cut-off points for the lowest quintile of calf circumference/BMI: <1.31 in men or <1.21 in women.

### Muscle function

Low muscle strength was defined as either low knee extension strength (KES) or slow gait speed [47]. KES was measured isometrically in the dominant leg, with the participant seated, the angles of the hip and knee at 90°, using Lord’s strap and strain gauge assembly component of the Physiological Profile Assessment (PPA) [48]. The average value (in kg) of three trials was estimated. Low KES was defined as the lowest quintile of the values for the whole SLAS-II cohort, stratified by sex and BMI (Asian classification) as follows: (1) in men: underweight: <9.5 kg, normal weight: <12.3 kg, overweight: <14.7 kg, obese: <15.0 kg; (2) in women: underweight: <9.3 kg, normal weight: <10.0 kg, overweight: <10.0 kg, obese: <10.0 kg [49, 50].

### Gait speed

Gait speed was assessed using the average of 2 measurements of 6-m fast gait speed tests [51]. Participants whose gait speed was <0.8 m/s were classified as having slow gait speed – this translated to the lowest quintile of values for the whole cohort, adjusted by sex and height.

### Statistical analysis

Patient characteristics were summarized using median (IQR) for continuous variables and frequency (percentage) for categorical variables. Vascular indices and skeletal muscle metrics were summarized using median (IQR). The primary analyses were performed using linear regression which evaluated skeletal muscle metrics as dependent variables individually for their association with markers of carotid atherosclerosis, arterial stiffness, and endothelial function. Multivariable analyses were performed using hierarchical linear regression models that controlled for potential confounding variables: (i) model 1: adjusted for age, sex, and smoking status; (ii) model 2: adjusted for age, sex, smoking status, central obesity, diabetes, hypertension, dyslipidemia. We further analyzed for interaction by age and sex (considered significant if p<0.200) and performed stratified analyses, where applicable [52]. Results are presented as the coefficients (β) and p values, with statistical significance set at p <0.05 (two-tailed). All statistical analyses were carried out using STATA software (version 14.0).

## Supplementary Material

Supplementary Tables
