# Supplementary material for: Subclinical vasculopathy and skeletal muscle metrics in the singapore longitudinal ageing study
Source: Aging (Albany NY). 2021 Jun 7;13(11):14768–84. doi: 10.18632/aging.203142 (PMC8221343; doi:10.18632/aging.203142)
Supplement: Supplementary Tables [file aging-13-203142-s001.pdf]

SUPPLEMENTARY TABLES

Supplementary Table 1. Association between indices of aortic stiffness and skeletal muscle metrics (n=336).

		Gait speed			Knee strength			Calf circumference/BMI		
		β	p	p+	β	p	p+	β	p	p+
cfPWV	Univariate	-0.03	0.101		-0.44	0.223		-0.01	0.546	
	Multivariable	0.01	0.780		-0.54	0.179		-0.01	0.568	
	Multivariable (with age interaction)	0.02	0.425	0.205	-0.40	0.361	0.480	-0.01	0.512	0.742
	Age-stratified (< 70)	0.02	0.451		-0.54	0.324		-0.01	0.514	
	Age-stratified (≥ 70)	-0.04	0.296		-0.54	0.354		-0.00	0.864	
	Multivariable (with sex interaction)	0.02	0.483	0.350	-0.85	0.065	0.170	0.00	0.968	0.271
	Male	0.01	0.832		-0.62	0.275		0.01	0.291	
	Female	0.01	0.803		0.02	0.972		-0.04	0.083	
Composite z-score	Univariate	-0.08	0.003*		-1.52	0.004*		-0.03	0.093	
	Multivariable	-0.02	0.387		-0.40	0.466		-0.00	0.988	
	Multivariable (with age interaction)	-0.04	0.215	0.196	-0.01	0.989	0.481	0.01	0.720	0.738
	Age-stratified (< 70)	0.00	0.915		-0.03	0.967		-0.01	0.574	
	Age-stratified (≥ 70)	-0.17	0.017*		-1.75	0.141		0.02	0.537	
	Multivariable (with sex interaction)	-0.04	0.268	0.426	-0.11	0.870	0.168	0.01	0.626	0.250
	Male	-0.06	0.184		-0.80	0.365		0.03	0.105	
	Female	0.02	0.635		0.35	0.575		-0.03	0.252	

Association between indices of aortic stiffness and skeletal muscle metrics, where arterial path length (for cfPWV) was derived using population-derived formula.

Univariate, multivariable, age-stratified and sex-stratified analyses were carried out. Multivariable analysis was performed using linear regression, accounting for demographics (age, sex) and cardiometabolic risk factors (smoking, central obesity, diabetes, hypertension and dyslipidemia). Age-stratified analysis of the associations between aortic stiffness and skeletal muscle metrics, accounting for demographics (sex) and cardiometabolic risk factors (smoking, central obesity, diabetes, hypertension and dyslipidemia). Sex-stratified analysis of the associations between aortic stiffness and skeletal muscle metrics, accounting for demographics (age) and cardiometabolic risk factors (smoking, central obesity, diabetes, hypertension and dyslipidemia).

p , p-value for vascular marker; p+, p-value for interaction of vascular marker and age, or interaction of vascular marker and sex.

*Statistically significant at p<0.05.

Abbreviations: cfPWV, carotid-femoral pulse wave velocity; Calf circumference/BMI, calf circumference adjusted for body mass index.

Supplementary Table 2. Multivariable analysis of subclinical vasculopathy and skeletal muscle metrics, accounting for interactions (n=336).

		Gait speed			Knee strength			Calf circumference/BMI		
		β	p^{+}	p^{++}	β	p^{+}	p^{++}	β	p^{+}	p^{++}
Atherosclerosis	cIMT	-0.26*	0.710	0.824	-3.28	0.233	0.121	0.04	0.840	0.122
Carotid stiffness#	AC	-0.03	0.803	0.661	0.49	0.685	0.933	-0.01	0.590	0.638
	Ep	-0.00	0.959	0.892	0.00	0.728	0.854	0.00	0.862	0.952
	β-index	-0.02	0.790	0.751	-0.06	0.719	0.700	0.00	0.631	0.948
	cAIx	0.01	0.260	0.493	0.21	0.410	0.809	0.00	0.830	0.410
	cPWV	-0.01	0.971	0.846	0.05	0.562	0.605	-0.00	0.691	0.771
	Composite z-score	-0.01	0.604	0.720	0.17	0.946	0.514	0.01	0.753	0.550
Aortic stiffness^	cfPWV	0.01	0.153	0.506	-0.59	0.472	0.206	-0.01	0.668	0.209
	aAix	-0.02	0.231	0.207	-0.19	0.453	0.176	0.00	0.009	0.555
	aPP	-0.01	0.722	0.411	0.11	0.913	0.471	-0.00	0.902	0.220
	Composite z-score	-0.03	0.191	0.468	-0.46	0.754	0.071	-0.00	0.191	0.166
Endothelial function	RHI	-0.01	0.307	0.289	-0.64	0.979	0.826	0.02	0.119	0.727

β shows the effect size for models adjusting for demographics (age, sex) and cardiometabolic risk factors (smoking, central obesity, diabetes, hypertension and dyslipidemia) without considering interaction terms.

p^{+}, p-value for vascular marker and age interaction; p^{++}, p-value for vascular marker and sex interaction.

#Indices of carotid stiffness calculated using derived central aortic pressures.

^Indices of aortic stiffness, where arterial path length was measured.

Abbreviations: cIMT, carotid intima-medial thickness; AC, arterial compliance; Ep, elastic modulus; β, beta; cAIx, carotid augmentation index; cPWV, carotid pulse wave velocity; cfPWV, carotid-femoral pulse wave velocity; aAIx, aortic augmentation index; aPP, aortic pulse pressure; RHI, reactive hyperemia index; Calf circumference/BMI, calf circumference adjusted for body mass index.

Supplementary Table 3. Age-stratified linear regression of subclinical vasculopathy and skeletal muscle metrics.

(A) Age ≥ 70 (n=56).

		Gait speed		Knee strength		Calf circumference/BMI	
		β	p	β	p	β	p
Atherosclerosis	cIMT	-0.48	*0.038	-9.33	*0.015	-0.01	0.956
Carotid stiffness#							
	AC	0.16	0.248	5.05	*0.031	-0.04	0.577
	Ep	-0.00	0.797	-0.01	0.152	-0.00	0.992
	β-index	-0.00	0.862	-0.55	0.217	0.00	0.980
	cAIx	-0.05	0.291	1.47	*0.045	-0.01	0.624
	cPWV	-0.00	0.922	-0.84	0.089	-0.00	0.920
	Composite z-score	-0.02	0.618	-0.65	0.386	-0.00	0.841
Aortic stiffness^	cfPWV	-0.05	0.128	-0.71	0.240	0.00	0.973
	aAIx	-0.11	*0.021	-0.43	0.585	0.05	0.051
	aPP	-0.06	0.290	-1.07	0.222	-0.01	0.609
	Composite z-score	-0.19	*0.007	-1.92	0.105	0.03	0.448
Endothelial function	RHI	-0.14	0.107	-0.82	0.566	0.02	0.612

(B) Age < 70 (n=280).

		Gait speed		Knee strength		Calf circumference/BMI	
		β	p	β	p	β	p
Atherosclerosis	cIMT	-0.20	0.103	-1.33	0.574	0.05	0.409
Carotid stiffness#							
	AC	-0.05	0.343	-0.10	0.927	-0.00	0.940
	Ep	-0.00	0.581	0.01	0.257	0.00	0.988
	β-index	-0.02	0.450	0.17	0.727	0.01	0.602
	cAIx	0.03	0.099	0.16	0.691	0.00	0.678
	cPWV	-0.00	0.932	0.52	0.253	-0.00	0.867
	Composite z-score	0.02	0.614	0.67	0.316	0.01	0.708
Aortic stiffness^	cfPWV	0.02	0.539	-0.53	0.310	-0.01	0.264
	aAIx	-0.00	0.976	-0.10	0.815	-0.01	0.516
	aPP	-0.01	0.752	0.34	0.390	0.00	0.965
	Composite z-score	0.00	0.949	-0.05	0.943	-0.01	0.442
Endothelial function	RHI	0.01	0.707	-0.53	0.485	0.02	0.394

Age-stratified analysis of the associations between subclinical vasculopathy and skeletal muscle metrics, accounting for demographics (sex) and cardiometabolic risk factors (smoking, central obesity, diabetes, hypertension and dyslipidemia)
*Statistically significant at p<0.05.
#Indices of carotid stiffness calculated using derived central aortic pressures.
^Indices of aortic stiffness, where arterial path length was measured.
Abbreviations: cIMT, carotid intima-medial thickness; AC, arterial compliance; Ep, elastic modulus; β, beta; cAIx, carotid augmentation index; cPWV, carotid pulse wave velocity; cfPWV, carotid-femoral pulse wave velocity; aAIx, aortic augmentation index; aPP, aortic pulse pressure; RHI, reactive hyperemia index; Calf circumference/BMI, calf circumference adjusted for body mass index.

Supplementary Table 4. Sex-stratified linear regression of subclinical vasculopathy and skeletal muscle metrics.

(A) Male (n=185).

		Gait speed		Knee strength		Calf circumference/BMI	
		β	p	β	p	β	p
Atherosclerosis	cIMT	-0.31	*0.028	-4.11	0.148	0.13	*0.019
Carotid stiffness[#]							
	AC	0.00	0.954	0.19	0.893	-0.02	0.526
	Ep	-0.00	0.355	0.01	0.621	0.00	0.826
	β-index	-0.03	0.333	0.26	0.655	0.01	0.647
	cAIx	0.00	0.912	0.26	0.632	-0.01	0.488
	cPWV	-0.01	0.689	0.29	0.597	-0.00	0.794
	Composite z-score	-0.03	0.516	0.69	0.415	-0.00	0.760
Aortic stiffness^	cfPWV	-0.00	0.746	-0.29	0.228	0.00	0.397
	aAIx	-0.04	0.149	-0.51	0.359	0.01	0.494
	aPP	-0.04	0.195	0.18	0.760	0.02	0.173
	Composite z-score	-0.07	0.107	-0.86	0.332	0.03	0.129
Endothelial function	RHI	0.03	0.496	-0.53	0.608	0.01	0.523

(B) Female (n=151).

		Gait speed		Knee strength		Calf circumference/BMI	
		β	p	β	p	β	p
Atherosclerosis	cIMT	-0.17	0.271	-0.30	0.908	-0.08	0.435
Carotid stiffness[#]							
	AC	-0.08	0.328	0.78	0.596	0.02	0.717
	Ep	-0.00	0.895	-0.01	0.384	-0.00	0.865
	β-index	-0.01	0.789	-0.56	0.137	0.00	0.932
	cAIx	0.02	0.303	0.35	0.392	0.01	0.541
	cPWV	-0.01	0.972	-0.38	0.340	-0.00	0.901
	Composite z-score	0.01	0.742	-0.46	0.424	0.01	0.787
Aortic stiffness^	cfPWV	0.01	0.757	-0.13	0.809	-0.04	0.050
	aAIx	0.00	0.988	0.26	0.536	-0.00	0.989
	aPP	0.02	0.477	0.18	0.653	-0.01	0.350
	Composite z-score	0.02	0.609	0.29	0.647	-0.03	0.196
Endothelial function	RHI	-0.03	0.565	-0.34	0.686	0.03	0.308

Sex-stratified analysis of the associations between subclinical vasculopathy and skeletal muscle metrics, accounting for demographics (age) and cardiometabolic risk factors (smoking, central obesity, diabetes, hypertension and dyslipidemia).
*Statistically significant at p<0.05.
[#]Indices of carotid stiffness calculated using derived central aortic pressures.
^Indices of aortic stiffness, where arterial path length was measured.
Abbreviations: cIMT, carotid intima-medial thickness; AC, arterial compliance; Ep, elastic modulus; β, beta; cAIx, carotid augmentation index; cPWV, carotid pulse wave velocity; cfPWV, carotid-femoral pulse wave velocity; aAIx, aortic augmentation index; aPP, aortic pulse pressure; RHI, reactive hyperemia index; Calf circumference/BMI, calf circumference adjusted for body mass index.
